# Ferroptosis in cancer therapy: a novel approach to reversing drug resistance

**DOI:** 10.1186/s12943-022-01530-y

**Published:** 2022-02-12

**Authors:** Chen Zhang, Xinyin Liu, Shidai Jin, Yi Chen, Renhua Guo

**Affiliations:** 1 Jiangsu Province Hospital and Nanjing Medical University First Affiliated Hospital, 300 Guangzhou Road, Nanjing, Jiangsu Province China; 2 Nanjing Pukou Central Hospital, Pukou Branch Hospital of Jiangsu Province Hospital, Nanjing, Jiangsu China

**Keywords:** Ferroptosis, Chemotherapy, Targeted therapy, Immunotherapy, Drug resistance

## Abstract

Ferroptosis is an intracellular iron-dependent form of cell death that is distinct from apoptosis, necrosis, and autophagy. Extensive studies suggest that ferroptosis plays a pivotal role in tumor suppression, thus providing new opportunities for cancer therapy. The development of resistance to cancer therapy remains a major challenge. A number of preclinical and clinical studies have focused on overcoming drug resistance. Intriguingly, ferroptosis has been correlated with cancer therapy resistance, and inducing ferroptosis has been demonstrated to reverse drug resistance. Herein, we provide a detailed description of the mechanisms of ferroptosis and the therapeutic role of regulating ferroptosis in reversing the resistance of cancer to common therapies, such as chemotherapy, targeted therapy and immunotherapy. We discuss the prospect and challenge of regulating ferroptosis as a therapeutic strategy for reversing cancer therapy resistance and expect that our review could provide some references for further studies.

## Background

Ferroptosis is an intracellular iron-dependent form of cell death that is distinct from apoptosis, necrosis, and autophagy. Although ferroptosis-like cell death has been observed in recent decades [1–3], only recently has this nonapoptotic form of cell death been termed “ferroptosis” by Dixon et al. [4], who found that the oncogenic RAS-selective lethal small molecule erastin activates a lethal pathway that is different from commonly regulated cell death. Erastin-induced cell death is also referred to as ferroptosis, and it is characterized by a redox state imbalance with increased levels of intracellular reactive oxygen species (ROS). It is well known that the loss of redox state balance in biological systems is responsible for many disorders [5]. Identified as a nexus linking redox biology and cell physiological functions, ferroptosis has been implicated in the pathological cell death associated with degenerative diseases, ischemic disorders and carcinogenesis [6].

In 2014, Yang et al. reported that renal cell carcinoma is particularly susceptible to ferroptosis and identified glutathione peroxidase 4 (GPX4) as a central regulator of ferroptosis [7]. GPX4 can catalyze the reduction of lipid peroxides, which plays a vital role in protecting against excessive lipid peroxidation [8]. GPX4-regulated ferroptosis inhibited tumor growth in a xenograft mouse model, and knockdown of GPX4 sufficiently killed renal cell carcinoma cell lines. In 2015, Jiang et al. found that inactivation of the p53 tumor suppression pathway in the formation of most human cancers was also associated with ferroptosis suppression [9]. Activation of p53 inhibited cystine uptake through the cystine/glutamate antiporter, which in turn limited the production of intracellular glutathione (GSH), thus protecting tumor cells from ferroptosis. Alvarez et al. also showed that resistance to ferroptosis is necessary for lung adenocarcinoma to survive in a high oxygen environment [10]. They discovered that the iron–sulfur cluster biosynthetic enzyme suppressing nitrogen fixation 1 (NFS1) is particularly important for maintaining iron-sulfur cofactors upon exposure to oxygen and protecting cells from undergoing ferroptosis. Recently, in 2020, Ubellacker et al. revealed that melanoma cells prefer to form more metastases through the lymphatic system than through the blood to avoid ferroptosis [11]. Due to the higher levels of GSH and oleic acid and less free iron in lymph, forming metastases in the lymphatic environment protects melanoma cells from ferroptosis and increases their ability to survive during subsequent metastasis through the blood. This evidence suggests that ferroptosis plays a pivotal role in tumor suppression and could be harnessed for cancer therapy.

Despite significant advances in oncological therapy, the development of resistance in tumors remains a major challenge. Sizable preclinical and clinical studies focused on overcoming drug resistance. Ferroptosis has also recently been proven to correlate with cancer therapy resistance. A high mesenchymal cell state in carcinomas has long been recognized to be associated with resistance to multiple cancer treatment modalities. Viswanathan et al. found that the therapy-resistant high-mesenchymal cell state depends on the GPX4-regulated lipid-peroxidase pathway that protects against ferroptosis [12]. In addition, numerous studies have indicated that the regulation of ferroptosis could influence the efficacy of cancer treatment and even reverse cancer therapy resistance [13–15]. Herein, we provide a detailed description of the mechanisms of ferroptosis and the therapeutic role of regulating ferroptosis in reversing cancer resistance to common therapies, such as chemotherapy, targeted therapy and immunotherapy. We discuss the prospect and challenge of regulating ferroptosis as a therapeutic strategy for reversing cancer therapy resistance and expect that our review could provide some references for further studies.

### Mechanisms of ferroptosis

Since the GPX4-centered mechanisms of ferroptosis were established in 2014 [7], an increasing number of studies have been conducted to identify novel mechanisms governing ferroptosis. GPX4-independent pathways also have been identified. These studies have offered a powerful theoretical framework for initiating the process of ferroptosis, which is briefly divided into the following pathways: the canonical GPX4-regulated pathway, iron metabolism pathway and lipid metabolism pathway.

#### Canonical GPX4-regulated pathway

**Fig. 1:**
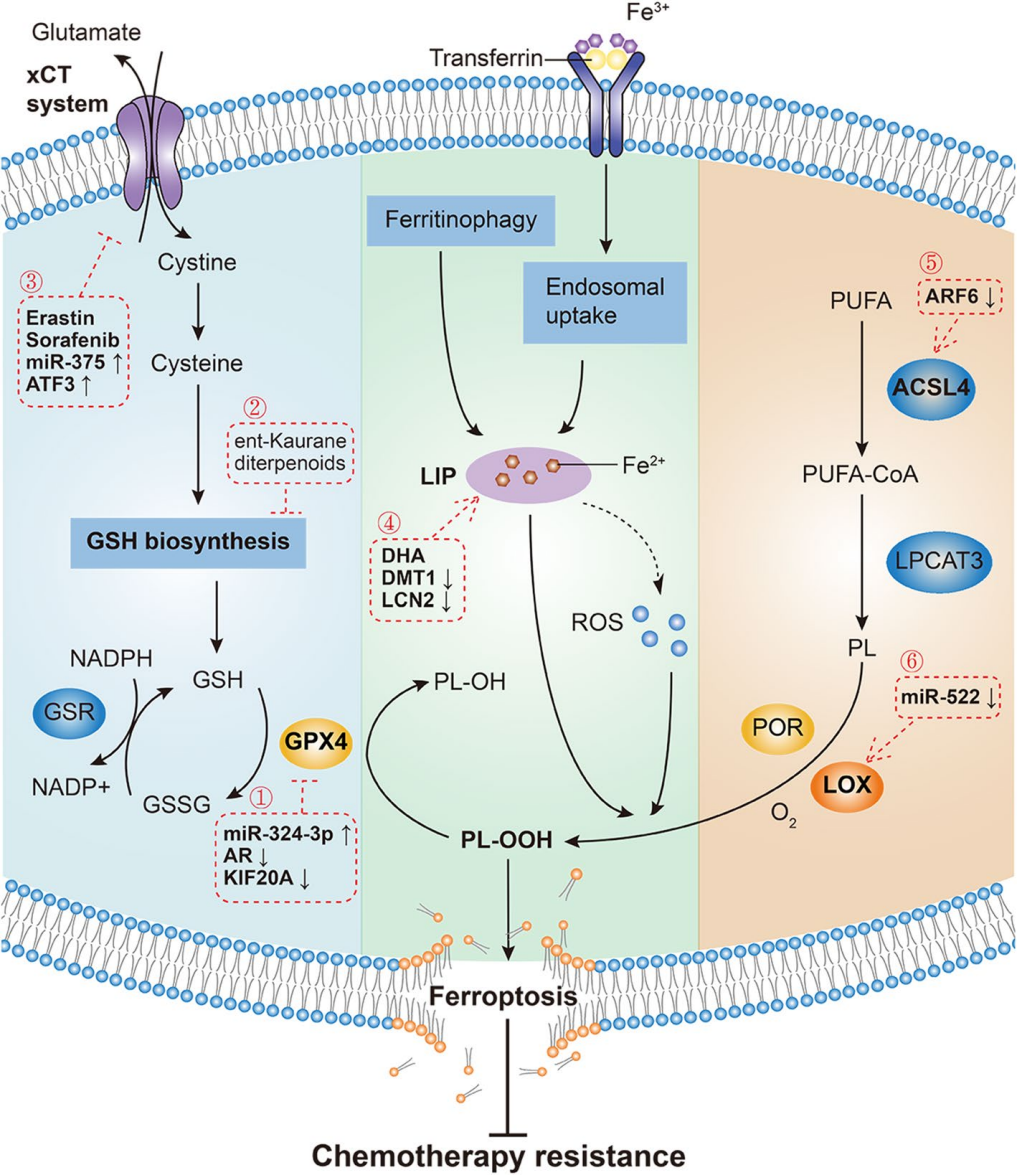
Mechanisms governing ferroptosis and reversing chemotherapy resistance. Three pathways initiate the process of ferroptosis and chemotherapy resistance reversal: the canonical GPX4-regulated pathway, iron metabolism pathway and lipid metabolism pathway. Regulation of the canonical GPX4-regulated pathway is as follows: ① Directly inhibit GPX4 by upregulating miR-324-3p or downregulating AR and KIF20A. ② Inhibit GSH biosynthesis by ent-kaurane diterpenoids. ③ Inhibit cystine uptake by erastin and sorafenib or upregulating miR-375 and ATF3. Regulation of the iron metabolism pathway is as follows: ④ Increase cellular LIP by DHA, downregulating DMT1 and LCN2. Regulation of the lipid metabolism pathway is as follows: ⑤ Target ACSL4 by downregulating ARF6. ⑥ Target LOX by downregulating miR-522. xCT: cystine/glutamate antiporter; ATF3: activating transcription factor 3; GSH: glutathione; GPX4: glutathione peroxidase 4; AR: androgen receptor; LIP: labile iron pool; DHA: dihydroartemisinin; DMT1: divalent metal transporter 1; LCN2: lipocalin 2; ROS: reactive oxygen species; PLOH: phospholipid alcohols; PLOOH: phospholipid hydroperoxides; PUFA: polyunsaturated fatty acid; ARF6: ADP ribosylation factor 6; ACSL4: acyl-CoA synthetase long chain family member 4; CoA: coenzyme A; LPCAT: lysophosphatidylcholine acyltransferase; PL: phospholipid; LOX: lipoxygenases; POR: cytochrome P450 oxidoreductase

Yang et al.’s study induced ferroptosis with different ferroptosis-inducing compounds (FINs) and found that all FINs inhibited GPX4 directly or indirectly through GSH depletion. They thus concluded that GPX4 is the key regulator of ferroptosis [7]. As a glutathione peroxidase in mammals, GPX4 appears to play a particularly important role in catalyzing the reduction of phospholipid hydroperoxides (PLOOH) into corresponding phospholipid alcohols [16]. GSH is necessary for the normal physiological function of GPX4 [17]. Intracellular GSH synthesis is catalyzed by glutamate–cysteine ligase. For this reason, cysteine, which is taken up as cystine via the cystine/glutamate antiporter (xCT) system, is referred to as the rate-limiting amino acid for the synthesis of GSH [18]. As a result, depriving cells of cysteine by inhibiting xCT system also contributes to the indirect inhibition of GPX4. Consequently, inactivation of GPX4 can lead to the accumulation of PLOOH, thus inducing cell membrane damage and ferroptotic death (Fig. 1). Earlier in 2008, inactivation of GPX4 was demonstrated to trigger redox-regulated cell death and cause neurodegeneration in vivo and ex vivo [19]. In Yang et al.’s study, it was also demonstrated that genetic inhibition of GPX4 can induce tumor cell ferroptosis and inhibit tumor growth in vivo [7]. These findings suggest that the canonical GPX4-regulated pathway plays an important role in tumor biology.

#### Iron metabolism pathway

As an iron-dependent form of cell death, ferroptosis is characterized by an increase in the small pool of Fe^2+^, which is referred to as the labile iron pool (LIP). In 1997, it was found that cellular iron uptake was mostly mediated by the binding of serum transferrin to the transferrin receptor and transferrin endocytosis [20]. In 2016, Wen et al. demonstrated that autophagy contributes to ferroptosis by degrading ferritin in fibroblasts and cancer cells. Overexpression of nuclear receptor coactivator 4 increased intracellular LIP by increasing ferritin degradation (namely ferritinophagy) [21]. On the one hand, the increased intracellular LIP can generate free radicals (hydroxyl radicals) through Fenton reaction and participate in the peroxidation of phospholipids to produce PLOOH [22]. On the other hand, the generation of most ROS in cells is iron-catalyzed. The generation of ROS initiates lipid peroxidation and eventually leads to ferroptosis [23]. Cancer cells have been shown to have increased iron requirements for survival in comparison to normal cells [24]. Iron uptake is enhanced and intracellular iron level is increased in rapidly proliferating cancer cells, shedding light on the potential of ferroptosis inducing as a therapeutic target for cancer. Alvarez et al. showed that increasing intracellular LIP by suppressing NFS1 sensitizes lung cancer cells to ferroptosis and reduces the growth of lung tumors in vivo [10]. In addition, Chang et al. found that BAY 11–7085, a well-known IκBα inhibitor, activates heme oxygenase-1 and enhances cancer cell ferroptosis by increasing LIP [25]. Taken together, the modulation of the iron metabolism pathway serves as a therapeutic means to trigger cancer cell ferroptosis.

#### Lipid metabolism pathway

The specific feature of ferroptosis is increased lipid peroxidation; thus, the metabolism of lipid peroxides is considered to be critical in the process of ferroptosis. Ferroptosis is likely performed by the peroxidation of membrane phospholipids to produce PLOOH and the decomposition of PLOOH to generate 4-hydroxynonenal or malondialdehyde. The products of lipid peroxidation cause membrane instability and permeabilization and eventually lead to cell death [26].

In nonenzymatic lipid peroxidation, polyunsaturated fatty acids (PUFAs) are ligated with coenzyme A (CoA) through the function of acyl-CoA synthetase long chain family member 4 (ACSL4) to produce acyl-CoA. Then, acyl-CoA can be re-esterified in phospholipids through various lysophosphatidylcholine acyltransferases (LPCATs) to produce PL. Therefore, the regulation of ACSL4 and LPCATs can dictate ferroptosis sensitivity [27, 28]. In enzymatic lipid peroxidation, PLOOH production can also be mediated by lipoxygenases (LOX) and cytochrome P450 oxidoreductase (POR). LOXs are nonheme iron-containing enzymes that directly catalyze the deoxygenation of free and esterified PUFAs to generate PLOOH [29]. A previous study demonstrated that overexpression of LOX-5, LOX-12, and LOX-15 sensitizes cells to ferroptosis. LOX inhibitors have also been demonstrated to be effective antioxidants that protect cells from lipid peroxidation [30]. In 2020, Zou et al. identified POR as necessary for ferroptotic cell death in cancer cells by genome-wide, CRISPR–Cas9-mediated suppressor screens [31]. Prior studies suggested that P450 could accept electrons from POR and catalyze the peroxidation of PUFAs [32]. The pro-ferroptotic role of POR has also been demonstrated by genetic depletion across a wide range of lineages and cell states [31].

### Reversing chemotherapy resistance by inducing ferroptosis

Many chemotherapy drugs have been found to induce ferroptosis (Table 1). The dysregulation of ferroptosis often leads to chemotherapy resistance and treatment failure. Pharmacological or genetic regulation of ferroptosis has been demonstrated to overcome chemotherapy resistance (Fig. 1). According to the mechanisms governing ferroptosis, there are three main pathways to reverse chemotherapy resistance, namely, the canonical GPX4-regulated pathway, iron metabolism pathway, and lipid metabolism pathway.

**Table 1:** Strategies and mechanisms of reversing drug resistance by inducing ferroptosis

**Reversing chemotherapy resistance by inducing ferroptosis.**					
**Chemotherapeutic Drugs**	**Ferroptosis-Inducing Compounds**	**Targets for Ferroptosis Induction**	**Cancer Type**	**Mechanisms**	**References**
Temozolomide	Curcumin analog	Glutathione peroxidase 4 (GPX4)	Glioblastoma	Curcumin analog induces androgen receptor (AR) ubiquitination and suppresses GPX4, thereby inducing ferroptosis and overcoming temozolomide resistance.	[33]
Oxaliplatin	/	GPX4	Colorectal cancer	Disrupting the KIF20A/NUAK1/PP1β/GPX4 pathway inhibits GPX4 to induce ferroptosis and overcomes oxaliplatin resistance.	[34]
Cisplatin	Ent-kaurane diterpenoids	Glutathione (GSH)	Lung cancer	Ent-kaurane diterpenoids target peroxiredoxin I/II and block GSH synthesis, thereby inducing ferroptosis and overcoming cisplatin resistance.	[35]
	Erastin and sulfasalazine	Cystine/glutamate antiporter (xCT)	Head and neck cancer	Erastin and sulfasalazine inhibit the xCT system to induce ferroptosis and overcome cisplatin resistance.	[36]
	/	xCT	Gastric cancer	Suppressing the Nrf2/Keap1/xCT pathway inhibits xCT system to induce ferroptosis and overcome cisplatin resistance.	[37]
	Dihydroartemisinin	Labile iron pool (LIP)	Pancreatic ductal adenocarcinoma	Dihydroartemisinin increases cellular LIP to induce ferroptosis and overcome cisplatin resistance.	[38]
5-fluorouracil	/	Lipocalin 2 (LCN2)	Colorectal cancer	Targeting LCN2 increases cellular LIP to induce ferroptosis and overcome 5-fluorouracil resistance.	[39, 40]
Multidrugs	/	Divalent metal transporter 1 (DMT1)	Breast cancer	Inhibition of DMT1 increases cellular LIP to induce ferroptosis and overcome multidrug resistance.	[41]
**Reversing targeted therapy resistance by inducing ferroptosis.**					
**Targeted Drugs**	**Ferroptosis-Inducing Compounds**	**Targets for Ferroptosis Induction**	**Cancer Type**	**Mechanisms**	**References**
Olaparib	Sulfasalazine	xCT	Ovarian cancer	Sulfasalazine suppresses SLC7A11 to induce ferroptosis and overcome olaparib resistance.	[42]
Cetuximab	β-elemene	GPX4	Colorectal cancer (KRAS-mutant)	β-elemene inhibits GPX4 to induce ferroptosis and overcome cetuximab resistance.	[43]
Gefitinib	/	GPX4	Breast cancer	Inhibition of GPX4 induces ferroptosis and overcomes gefitinib resistance.	[44]
Sorafenib	Trigonelline	NRF2	Hepatocellular carcinoma	Trigonelline inhibits NRF2 to induce ferroptosis and overcome sorafenib resistance.	[45]
Epidermal growth factor receptor-tyrosine kinase inhibitors (EGFR-TKIs)	Vorinostat	xCT	Lung cancer	Vorinostat inhibits xCT system to induce ferroptosis and overcome EGFR-TKIs resistance.	[46]
Sunitinib	Artesunate	GPX4	Renal cell carcinoma	Artesunate inhibits GPX4 to induce ferroptosis and overcome sunitinib resistance.	[47]

#### Regulation of canonical GPX4-regulated pathway

GPX4 functions in concert with GSH to catalyze the reduction of hydrogen peroxide and organic hydroperoxides to water or the corresponding alcohols, which plays a vital role in protecting against ferroptosis [7, 8]. Direct inhibition of GPX4 leads to ferroptotic cell death. Chen et al. showed that androgen receptor induces resistance to temozolomide treatment in glioblastoma. Androgen receptor ubiquitination induced by the curcumin analog was demonstrated to suppress GPX4, thereby inducing ferroptosis and reversing temozolomide resistance in glioblastoma [33]. Similar results were obtained by Yang et al., who found that highly expressed KIF20A was correlated with oxaliplatin resistance in colorectal cancer. Cellular ferroptosis may be induced by disrupting the KIF20A/NUAK1/PP1β/GPX4 pathway, thus overcoming the resistance of colorectal cancer to oxaliplatin [34]. Moreover, ferroptosis can also be triggered indirectly by blocking the synthesis of GSH, the cofactor of GPX4. Recently, ent-kaurane diterpenoids have been reported to be able to overcome cisplatin resistance by inducing ferroptosis. Further mechanistic studies have revealed that ferroptosis induced by ent-kaurane diterpenoids is caused by targeting peroxiredoxin I/II and depleting GSH [35]. Cysteine is an essential cellular antioxidant and the rate-limiting amino acid for GSH biosynthesis. The xCT system mediates the uptake of cystine, which is rapidly reduced to cysteine, thus affecting GSH synthesis. Inhibition of the xCT system has also been shown to be capable of triggering ferroptosis. A study by Roh et al. revealed that xCT system inhibitors (erastin and sulfasalazine) or genetic silencing of the xCT system could trigger head and neck cancer cell ferroptosis and overcome cisplatin resistance [36]. Similarly, Fu et al. showed that inducing ferroptosis by restraining Nrf2/Keap1/xCT signaling also sensitized cisplatin-resistant cells to cisplatin in gastric cancer [37].

#### Regulation of iron metabolism pathway

Ferroptosis is defined as an iron-catalyzed form of regulated necrosis [38]. Elevated levels of cellular LIP increase vulnerability to ferroptosis. Du et al. reported that dihydroartemisinin treatment could overcome cisplatin resistance in pancreatic ductal adenocarcinoma by inducing ferroptosis. Further study revealed that tumor cell ferroptosis contributed to catastrophic accumulation of LIP after dihydroartemisinin treatment [38]. Lipocalin 2 (LCN2) is a secreted glycoprotein and may regulate iron homeostasis [39]. A recent study showed that LCN2 overexpression was correlated with 5-fluorouracil resistance in colon cancer. Targeting LCN2 overcame 5-fluorouracil resistance by increasing intracellular iron levels, which in turn led to tumor cell ferroptosis [40]. Divalent metal transporter 1 (DMT1) is another key protein in the regulation of iron homeostasis. Turcu et al. reported that increasing cellular LIP caused by DMT1 inhibition triggered ferroptosis, thus killing breast cancer stem cells and reversing multidrug resistance [41].

#### Regulation of lipid metabolism pathway

Accumulation of PLOOH is the hallmark of ferroptosis. Lipid metabolism is closely related to cell vulnerability to ferroptosis. PLOOH can accumulate in both enzymatic and nonenzymatic ways. Dull et al.’s study revealed that ACSL4 is an essential component for ferroptosis execution. ACSL4 functions in converting long chain-PUFAs to acyl-CoA, subsequently participating in producing PLOOH in an enzymatic way [27]. Ye et al. reported that the activation of ACSL4 by inhibiting ADP ribosylation factor 6 (ARF6) overcame gemcitabine resistance in pancreatic cancer by inducing ferroptosis [48]. In addition, several LOXs have been reported to be able to oxygenate PUFAs directly in a nonenzymatic way, thereby mediating ferroptosis [49]. Zhang et al. showed that arachidonate lipoxygenase 15 (ALOX15), a LOX member, was closely linked with the suppression of ferroptosis in gastric cancer. Downregulation of microRNA-522, which promotes ALOX15 expression, provides novel methods to enhance cisplatin/paclitaxel sensitivity in gastric cancer by inducing ferroptosis [50].

### Reversing resistance to targeted therapy by inducing ferroptosis

**Fig. 2:**
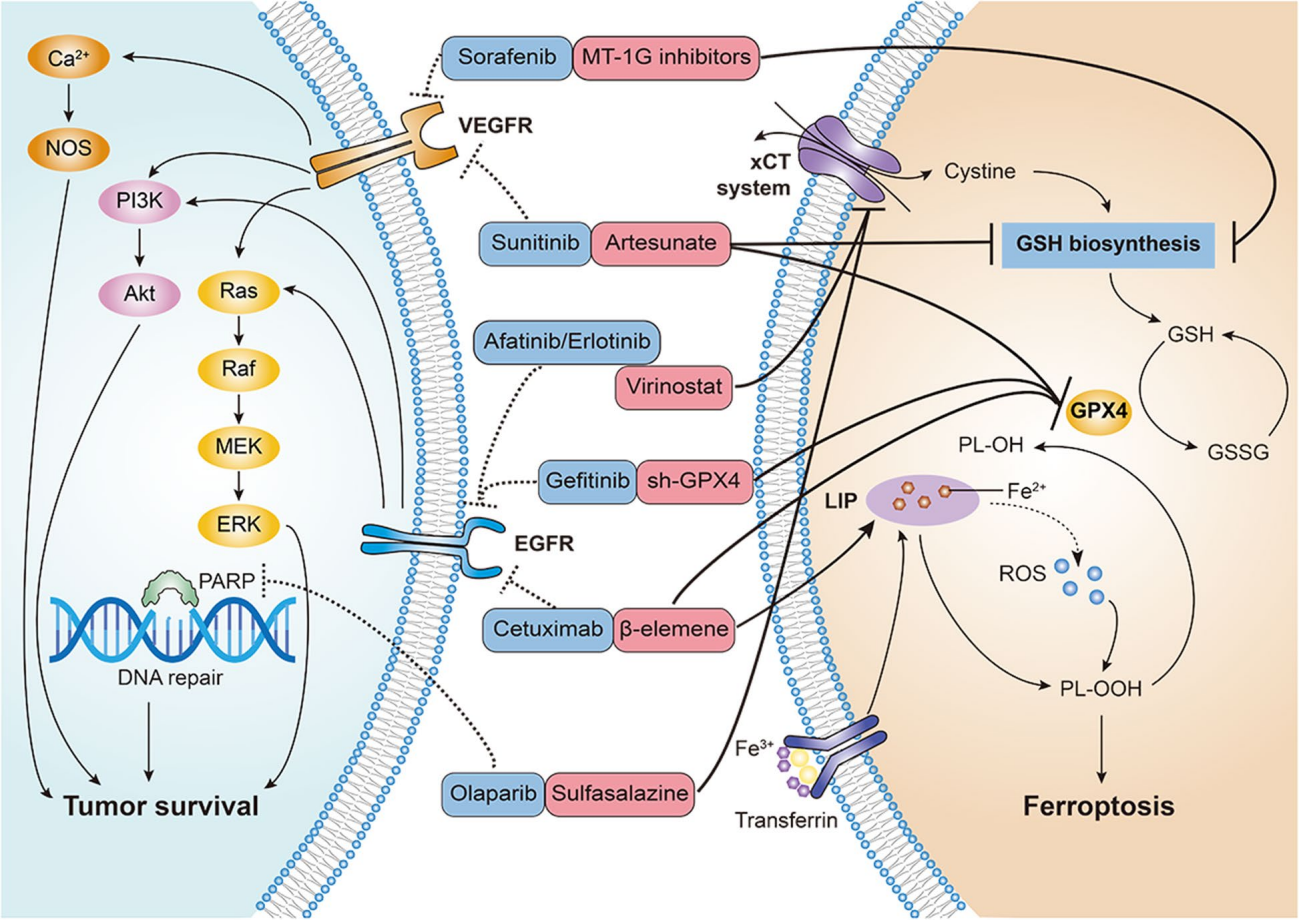
Reversing targeted therapy resistance by inducing ferroptosis. Activation of the downstream pathways of VEGFR, EGFR and PARP facilitates tumor survival and resistance to certain inhibitors (blue). Combination therapy with ferroptosis inducers (red) has been demonstrated to reverse targeted therapy resistance. VEGFR, vascular endothelial growth factor receptor; EGFR, epidermal growth factor receptor; PARP, poly (ADP-ribose) polymerase; xCT: cystine/glutamate antiporter; GSH: glutathione; GPX4: glutathione peroxidase 4; LIP: labile iron pool; ROS: reactive oxygen species; PLOH: phospholipid alcohols; PLOOH: phospholipid hydroperoxides

Acquired resistance to targeted therapy is common; thus, strategies for the management of drug resistance are urgently required. Emerging evidence suggests that ferroptosis plays a pivotal role in cancer therapy by interfering with target molecules and is involved in cancer development. Moreover, it has been reported ferroptosis can be used to overcome resistance to targeted therapy (Table 1, Fig. 2).

#### Ferroptosis in intrinsic resistance

##### Olaparib

Olaparib, a well-known poly (ADP-ribose) polymerase (PARP) inhibitor, has been approved for treatment and maintenance in advanced ovarian cancer patients with germline BRCA1/2 mutations by the United States Food and Drug Administration. However, patients who carry proficient BRCA (without germline BRCA mutations) do not benefit from olaparib. Solute carrier family 7 member 11 (SLC7A11) is a catalytic subunit of xCT system, functions as an important regulator of the cellular ferroptosis defense system and promotes cysteine supply and GSH biosynthesis. It has been reported that PARP inhibition can promote ferroptosis by suppressing SLC7A11-mediated GSH synthesis. Enhanced ferroptosis by FINs synergistically sensitizes BRCA-proficient ovarian cancer cells and xenografts to the PARP inhibitor olaparib [42]. This study reveals that the combination of PARP inhibitors and FINs might be a novel strategy for BRCA-proficient ovarian cancer.

##### Cetuximab

RAS mutations are present in approximately half of all metastatic colorectal cancer, and they greatly limit the therapeutic efficacy of anti-epidermal growth factor receptor (EGFR) antibodies, such as cetuximab. β-elemene, a natural product isolated from the Chinese herb Curcumae Rhizoma, is recommended as a new ferroptosis inducer. Combined treatment with β-elemene and cetuximab is sensitive to KRAS mutant metastatic colorectal cancer cells by inducing ferroptosis and inhibiting epithelial-mesenchymal transformation, which will provide a prospective therapeutic strategy for metastatic colorectal cancer patients with RAS mutations [43].

##### Gefitinib

Although clinical trials show that a wide range of tumor types benefit from gefitinib, triple-negative breast cancer cells are resistant to clinical doses of gefitinib. Inhibition of GPX4 promoted gefitinib sensitivity by promoting cell ferroptosis. Malondialdehyde and ROS production were increased after silencing GPX4, while the GSH levels were decreased [44]. GPX4 is a critical regulator and promising therapeutic target of gefitinib resistance in human triple-negative breast cancer cells.

#### Ferroptosis in acquired resistance

##### Sorafenib

Sorafenib has been identified as an inhibitor of multiple oncogenic kinases and is the first approved systemic therapy for patients with advanced hepatocellular carcinoma. However, its use is limited by acquired resistance, resulting in poor prognosis. Metallothioneins are small cysteine-rich proteins that play important roles in oxidative stress. Metallothionein-1G (MT-1G) is one of functional isoforms in metallothionein-1. Activation of the transcription factor nuclear factor erythroid 2-related factor 2 (NRF2) is essential for the induction of MT-1G expression after sorafenib treatment. MT-1G upregulation could limit GSH depletion and lipid peroxidation, which contributes to sorafenib-induced ferroptosis. Inhibition of MT-1G expression enhances the anticancer activity of sorafenib by ferroptosis induction both in vitro and in vivo [45]. This finding suggests that MT-1G is a new regulator of ferroptosis in hepatocellular carcinoma and demonstrates a novel molecular mechanism of sorafenib resistance.

##### EGFR-TKIs

EGFR is the most common driver mutation in lung adenocarcinoma, with an incidence of 37.3% in China. EGFR tyrosine kinase inhibitors (EGFR-TKIs) are the first approved first-line treatment for these patients. However, acquired resistance invariably develops, which becomes a clinical challenge. After developing acquired resistance to EGFR-TKIs, EGFR-mutant lung cancer cells display higher sensitivity to ferroptosis inducers. The histone deacetylase inhibitor vorinostat could promote ferroptosis in EGFR-mutant lung adenocarcinoma cells by inhibiting SLC7A11 (xCT) expression and enhancing the efficacy of ferroptosis inducers [46]. The results show certain therapeutic prospects in overcoming resistance to EGFR-TKIs.

##### Sunitinib

The TKI sunitinib extends the long progression-free survival of patients with renal cell carcinoma, although resistance occurs during treatment and limits its efficacy. Artesunate, a drug originating from traditional Chinese medicine, inhibits the growth of sunitinib-resistant renal cell carcinoma cells through cell cycle arrest and the induction of ferroptosis. GSH significantly decreased after treatment with artesunate compared to the untreated controls. The application of artesunate resulted in a significant reduction in GPX4 in drug-resistant cells [47].

Of note, these studies had several limitations. Most experiments were limited to in vitro scenarios and further investigations should be performed in vivo to verify the postulates. Meanwhile, limited panel of cell lines were analyzed. However, we are positive to suggest that these findings provide new insight into how cancer cells induce the ferroptotic cell death process and inducing ferroptosis therapeutic strategies can be developed to overcome resistance to targeted therapy.

### Reversing immunotherapy resistance by inducing ferroptosis

Immunotherapy with immune checkpoint inhibitors is a significant oncological breakthrough over the past decade and has shown promising efficacy in various malignancies. Despite the demonstrated clinical benefits of immune checkpoint inhibitors treatment, drug resistance remains a great challenge. Known resistance mechanisms of immunotherapy can be divided into tumor cell-intrinsic factors and tumor cell-extrinsic factors [51]. Tumor cell-intrinsic factors indicate the characteristics of tumor cells themselves, which prevent immune cell infiltration or function within the tumor microenvironment (TME). Tumor cell-extrinsic factors refer to other components of the TME beyond tumor cells that inhibit antitumor immune responses.

**Fig. 3:**
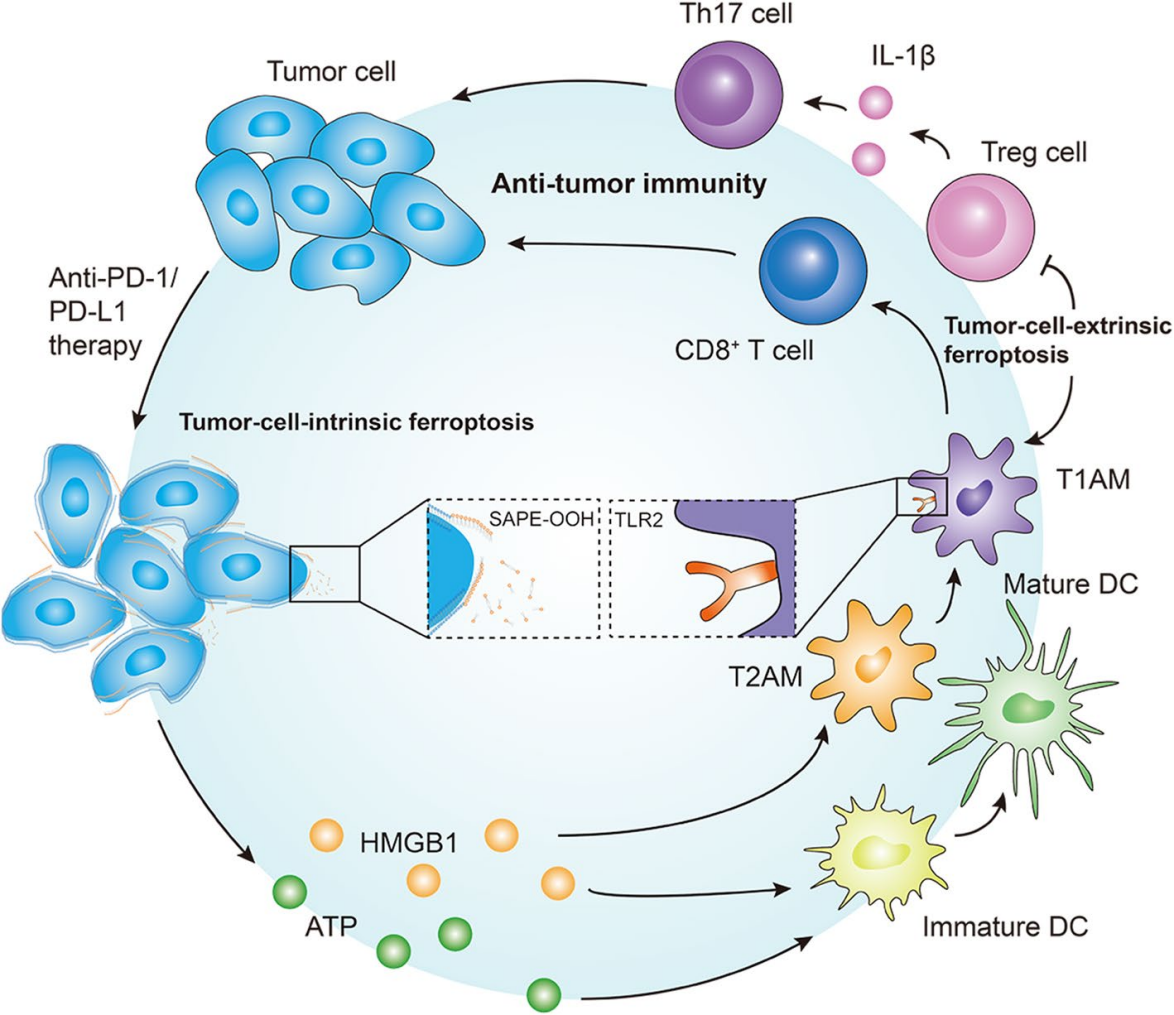
Reversing immunotherapy resistance by inducing ferroptosis. There are two approaches to reversing immunotherapy resistance by inducing ferroptosis: a tumor cell-intrinsic approach that induces ferroptosis in cancer cells to elicit a vaccination-like effect to stimulate antitumor immunity and a tumor cell-extrinsic approach that triggers ferroptosis in the TME to deplete immune suppressor cells. TME, tumor microenvironment; PD-1: programmed cell death 1; PD-L1: programmed death-ligand 1; SAPE-OOH: 1-steaoryl-2-15-HpETE-sn-glycero-3-phosphatidylethanolamine; Tregs: regulatory T cells; TAMs: tumor-associated macrophages; IL-1β: interleukin-1β; Th17: T helper 17; DC: dendritic cell; ATP: adenosine triphosphate; HMGB1: high-mobility group box 1

Recently, it has been shown that ferroptosis is involved in T cell immunity and cancer immunotherapy [52]. Jiang et al. reported that suppression of ferroptosis contributed to anti-programmed cell death 1 (PD-1)/programmed death-ligand 1 (PD-L1) therapy resistance [53]. These findings indicate that the induction of ferroptosis may overcome immunotherapy resistance (Fig. 3).

#### Tumor-cell-intrinsic mechanisms

Several tumor cell-intrinsic mechanisms leading to immunotherapy resistance have been identified, including the activated mitogen-activated protein kinase (MAPK) signaling pathway, loss of PTEN expression, expression of the WNT/β-catenin signaling pathway, loss of interferon-gamma signaling pathway, and loss of tumor antigen expression [51]. These changes result in the inability to mount potent antitumor immune responses. Recent studies have proposed that stimulating the adaptive immune system by causing immunogenic cell death may turn the immunologically cold state into a checkpoint blockade responsive state [54, 55]. Interestingly, ferroptosis has been demonstrated to be immunogenic. Efimova et al. described a novel approach to stimulate the adaptive immune system by triggering ferroptosis-dependent immunogenic cell death in preclinical models. Early ferroptotic cells have been reported to release damage-associated molecular patterns (adenosine triphosphate and high-mobility group box 1) and promote the phenotypic maturation of bone marrow-derived dendritic cells [56, 57]. In addition, Luo et al. identified an eat-me signal on the ferroptotic cancer cell surface, 1-steaoryl-2-15-HpETE-sn-glycero-3-phosphatidylethanolamine (SAPE-OOH). Moreover, enrichment of SAPE-OOH facilitated phagocytosis by targeting toll-like receptor 2 on macrophages [58]. Taken together, inducing ferroptosis in cancer cells may elicit a vaccination-like effect to stimulate antitumor immunity, thus overcoming immunotherapy resistance.

#### Tumor-cell-extrinsic mechanisms

Immune suppressor cells within the TME also contribute to resistance to immunotherapy, such as regulatory T cells (Tregs) and tumor-associated macrophages (TAMs) [51].

Quezada et al. reported that the effect of anti-cytotoxic T-lymphocyte antigen 4 (CTLA-4) immunotherapy was related to the ratio of effectors T cells to Tregs in the TME [59]. Tregs exert inhibitory control over infiltrating effectors T cells. In this study, combination with GM-CSF–transduced tumor cell vaccine potentiates the efficacy of CTLA-4 blockade by increasing the ratio of effectors T cells to Tregs. Another study by Oweida et al. also demonstrated that Tregs infiltration was associated with anti-PD-L1 immunotherapy resistance. Targeting Tregs depletion has been shown to restore antitumor immunity [60]. Notably, a recent study showed that GPX4 protects Tregs from ferroptosis. GPX4-deficient Tregs produce interleukin-1βand facilitate the T helper 17 response, which enhances antitumor immunity [61]. Taken together, these results indicate that inducing Tregs ferroptosis by suppressing GPX4 may reverse immunotherapy resistance.

As another subset of cells that seem to affect responses to immunotherapy, TAMs can polarize to two major phenotypes: antitumor M1 (TAM1) and protumor M2 (TAM2). TAM2 is often the dominant subset of TAMs in TME. Zhu et al. demonstrated that reprogramming TAMs by blocking CSF1/CSF1R enhanced the efficacy of immune checkpoint inhibitors in pancreatic cancer [62]. A recent study indicated that TAM1, which leads to higher levels of inducible nitric oxide (NO) synthase (iNOS)/NO•, exerted higher resistance to ferroptosis than TAM2. Regulation of ferroptosis by iNOS/NO• inhibited TAM2 survival without affecting TAM1, thus potentiating antitumor immunity in the TME [63]. Additionally, Jiang et al. showed that high TYRO3 expression was associated with anti-PD-1/PD-L1 immunotherapy resistance in a preclinical mouse model and in patients. TYRO3 inhibited tumor cell ferroptosis and facilitated the polarization of TAM1 to TAM2. Inhibition of TYRO3 also triggered ferroptosis and reprogrammed TAMs, thus resensitizing cancer cells to immunotherapy [53].

Collectively, triggering ferroptosis in the TME may be a tumor cell-extrinsic approach to overcoming immunotherapy resistance by depleting immune suppressor cells.

## Conclusions and perspectives

Ferroptosis is a form of programmed cell death in addition to apoptosis driven by iron-dependent phospholipid peroxidation, featuring the accumulation of ROS and lipid peroxidation overproduction [7]. Targeting the cell death process is a common method in cancer treatment, and ferroptosis is also considered to play a significant role in anticancer therapies recently. Intrinsic and acquired resistance are the main obstacles in cancer treatment. It has been reported that tumor cells may significantly enhance their oxidative stress defense ability by negatively regulating ferroptosis, thus leading to resistant survival [64], which shed light on reversing cancer resistance by inducing ferroptosis. As many studies have shown, regulation of ferroptosis may overcome resistance to traditional chemotherapy, targeted therapy and immunotherapy. These findings hold promises for the development of novel therapeutics by inducing ferroptosis for overcoming drug resistance in cancer.

In this review, we summarize the mechanisms of ferroptosis in reversing drug resistance in preclinical studies. However, there is still a long way to go before the practical application. Firstly, it is difficult to determine whether the reversing drug resistance role of ferroptosis inducers is only specific to certain cancer with unique characteristics or universally to most other cancers. Given the sensitivity to ferroptosis inducers varies greatly across cancer cell lines [65], we need to define an appropriate target population that is most likely to benefit from this strategy. Deeper insight into the mechanisms involved in ferroptosis and drug resistance will contribute to this goal. Secondly, ferroptosis has been implicated in the pathological cell death associated with multiple diseases besides cancer, such as degenerative diseases and ischemic disorders [6]. It will be particularly important to develop specific therapeutics inducing ferroptosis in cancer cells while avoiding systemic adverse effects. In this respect, nanoparticle ferroptosis inducers provide unique advantages [66]. Last but not least, we lack the biomarkers for labelling ferroptosis in vivo. Exploring suitable biomarkers will advance development of further in vivo researches and clinical monitoring.

## Data Availability

Not applicable.

## Abbreviations

ROS: Reactive oxygen species
GPX4: Glutathione peroxidase 4
GSH: Glutathione
NFS1: Nitrogen fixation 1
FINs: Ferroptosis-inducing compounds
PLOOH: Phospholipid hydroperoxides
xCT: Cystine/glutamate antiporter
LIP: Labile iron pool
PUFAs: Polyunsaturated fatty acids
CoA: Coenzyme A
ACSL4: Acyl-CoA synthetase long chain family member 4
LPCATs: Lysophosphatidylcholine acyltransferases
LOX: Lipoxygenases
POR: Cytochrome P450 oxidoreductase
LCN2: Lipocalin 2
DMT1: Divalent metal transporter 1
ARF6: ADP ribosylation factor 6
ALOX15: Arachidonate lipoxygenase 15
PARP: Poly (ADP-ribose) polymerase
SLC7A11: Solute carrier family 7 member 11
EGFR: Epidermal growth factor receptor
MT-1G: Metallothionein-1G
NRF2: Nuclear factor erythroid 2-related factor 2
TKI: Tyrosine kinase inhibitor
TME: Tumor microenvironment
PD-1: Programmed cell death 1
PD-L1: Programmed death-ligand 1
MAPK: Mitogen-activated protein kinase
SAPE-OOH: 1-steaoryl-2-15-HpETE-sn-glycero-3-phosphatidylethanolamine
Tregs: Regulatory T cells
TAMs: Tumor-associated macrophages
CTLA-4: Cytotoxic T-lymphocyte antigen 4
NO: Nitric oxide
iNOS: Inducible nitric oxide synthase
